# Supplementary material for: Direct Powder Extrusion of Paracetamol Loaded Mixtures for 3D Printed Pharmaceutics for Personalized Medicine via Low Temperature Thermal Processing
Source: Pharmaceutics. 2021 Jun 19;13(6):907. doi: 10.3390/pharmaceutics13060907 (PMC8234073; doi:10.3390/pharmaceutics13060907)
Supplement: Supplementary file 1 [file pharmaceutics-13-00907-s001.zip › pharmaceutics-1249643-supplementary.pdf]

pharmaceutics MDPI

Supplementary Materials: Direct Powder Extrusion of Paracetamol Loaded Mixtures for 3D Printed Pharmaceutics for Personalized Medicine via Low Temperature Thermal Processing

Xabier Mendibil, Gaizka Tena, Alaine Duque, Nerea Uranga, Miguel Ángel Campanero and Jesús Alonso

Table S1. Percentual mass losses of the individual constituents attributable to the moisture.

Mass Loss	
Material	Loss [%]
Guar Gum	6
Starch	10
Parecetamol	0
HPC	0

Table S2. Recovery percentages of each API containing mixture at different Guar gum content.

Guar Gum 0%wt				Guar Gum 5%wt				Guar Gum 10%wt			
Sample	Mean [%]	SD	CV	Sample	Mean [%]	SD	CV	Sample	Mean [%]	SD	CV
F05702500A	103	2	2	FG05652505A	104	2	3	FG05602510A	86	9	5
F20552500A	98	5	3	FG20502505A	96	2	5	FG20452510A	84	1	11
F35402500A	80	9	10	FG35352505A	79	5	6	FG35302510A	81	6	2
F50252500A	87	4	4	FG50202505A	96	7	7	FG50152510A	72	8	11

Table S3. Release curve of extruded mixtures containing 5% wt of Guar gum and different proportions of paracetamol and commercial aracetamol tablet at different sampling times.

FG05702505A				FG20552505A				FG35402505A				FG50252505A			
Time [min]	Mean [%]	SD	CV	Time [min]	Mean [%]	SD	CV	Time [min]	Mean [%]	SD	CV	Time [min]	Mean [%]	SD	CV
0	5	1	29	0	3	1	22	0	3	1	26	0	2	0	15
5	23	0	2	5	21	1	2	5	19	2	12	5	18	2	9
10	37	1	3	10	34	2	3	10	35	4	11	10	30	1	2
15	49	3	5	15	46	3	4	15	44	5	11	15	40	4	4
30	83	6	7	30	77	6	5	30	75	5	5	30	67	2	7
60	109	1	1	60	102	1	5	60	104	1	4	60	95	3	7

Table S4. Release curve of commercial paracetamol tablet at different sampling times.

Paracetamol			
**Time [min]**	**Mean [%]**	**SD**	CV
0	1	1	48
5	78	8	10
10	98	1	1
15	98	1	1
30	99	1	0

Table S5. Recovery percentages of samples containing 5% of Guar gum tested in fresh condition and stored for 6 weeks at room temperature.

	**Fresh Conditions**		Aged Conditions	
Sample	Mean [%]	SD	Mean [%]	SD
FG05702505A	88	3	80	1
FG20552505A	90	0	73	2
FG35402505A	93	2	74	3
FG50252505A	98	2	74	3

Table S6. Recovery percentages of preliminary tests with water quantity proportional to excipient amount instead to starch amount.

**Sample**	**Mean [%]**	**SD**
FG05702503	98	3
FG20552503	96	4
FG35402503	N/A	-
FG50252503	N/A	-

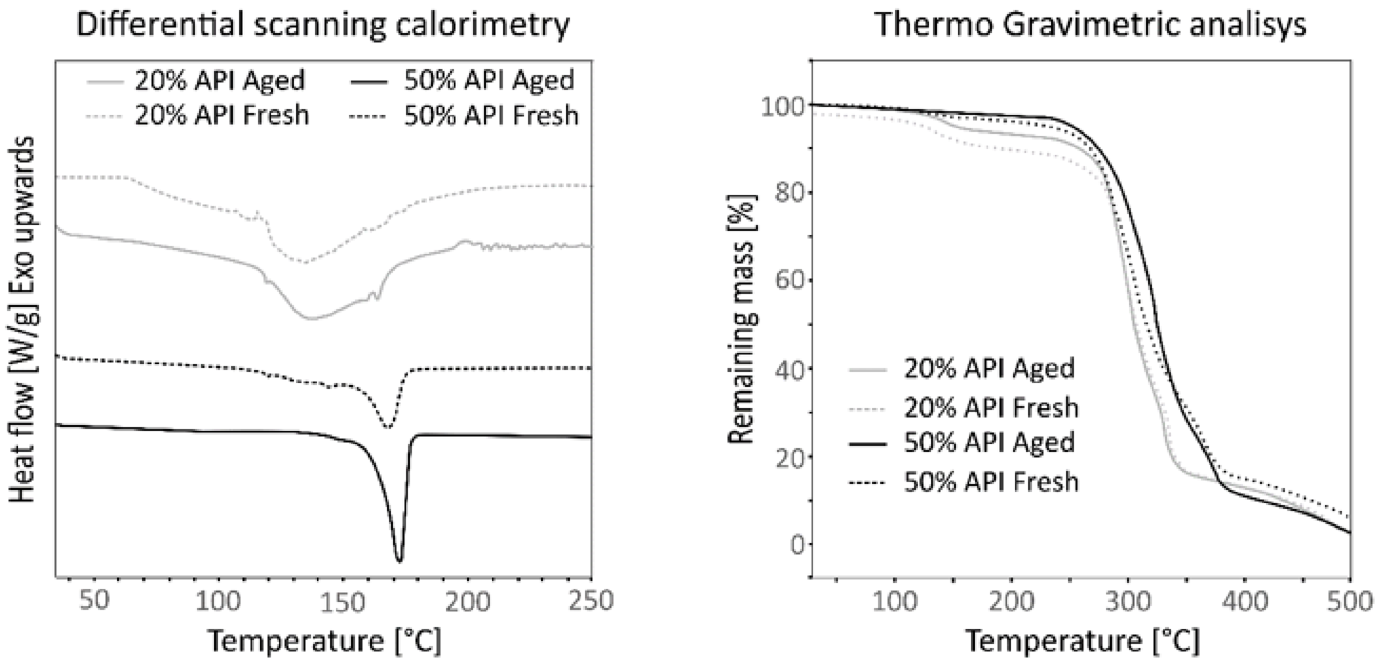

Figure S1. Differential scanning calorimetry (left panel) and thermogravimetric analysis (right panel) of samples containing 5% wt of Guar gum and 20% wt (grey) and 50% wt (black) of paracetamol in freshly extruded condition (dashed line) and in aged for 6 weeks condition (continuous line).

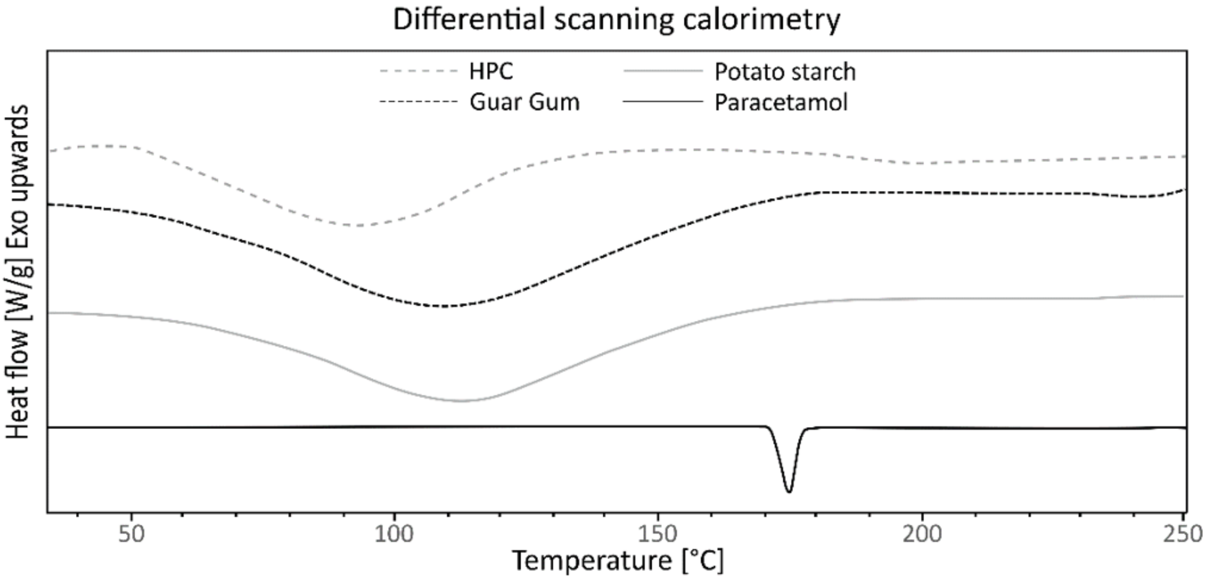

Figure S2. Differential scanning calorimetry of all the constituents of the mixture. HPC (dashed grey), Guar gum (dashed black), potato starch (continuous grey) and paracetamol (continuous black).
